# Pharmacological Potential and Electrochemical Characteristics of *Typha angustifolia* Pollen

**DOI:** 10.3390/plants13202857

**Published:** 2024-10-12

**Authors:** Janielle Mari S. Abadilla, Bor-Yann Chen, Mike Anthony D. Ganzon, Alvin R. Caparanga, Kristopher Ray S. Pamintuan, Lemmuel L. Tayo, Chung-Chuan Hsueh, Cheng-Yang Hsieh, Ling-Ling Yang, Po-Wei Tsai

**Affiliations:** 1 School of Chemical, Biological, and Materials Engineering and Sciences, Mapúa University, Manila 1002, Philippines; jmsabadilla@mymail.mapua.edu.ph (J.M.S.A.); madganzon@mymail.mapua.edu.ph (M.A.D.G.); arcaparanga@mapua.edu.ph (A.R.C.); krspamintuan@mapua.edu.ph (K.R.S.P.); lltayo@mapua.edu.ph (L.L.T.); 2 School of Graduate Studies, Mapúa University, Manila 1002, Philippines; 3 Department of Chemical and Materials Engineering, National I-Lan University, Yilan 260, Taiwan; boryannchen@yahoo.com.tw (B.-Y.C.); cchsueh@niu.edu.tw (C.-C.H.); d339108001@tmu.edu.tw (C.-Y.H.); 4 Department of Biology, School of Health Sciences, Mapúa University, Makati 1200, Philippines; 5 Department of Food Science, National Taiwan Ocean University, Keelung 202, Taiwan; 6 School of Pharmacy, College of Pharmacy, Taipei Medical University, Taipei 110, Taiwan; llyang@tmu.edu.tw; 7 Department of Acupuncture, American College of Acupuncture & Oriental Medicine, Houston, TX 77063, USA

**Keywords:** antiviral, electron shuttles, flavonoids, microbial fuel cells, *Typha angustifolia*

## Abstract

*Typha angustifolia* L. (TA) pollen has been utilized as a traditional Chinese medicine for treating various internal and external traumas. Moreover, bioactive compounds possess diverse pharmacological activities. This study aims to evaluate the antiviral properties of TA based on its ability to generate bioenergy, capable of inhibiting viruses. TA pollens were extracted using water and ethanol solvents. These extracts were utilized to identify the phytochemical contents and correlate with the antioxidant activity via 2,2-diphenyl-1-picrylhydrazyl (DPPH) and ferric reducing antioxidant power (FRAP) assays. HPLC analysis was conducted to identify its electron-shuttling compositions. The bioenergy-generating characteristics were determined via microbial fuel cells. The water extract (TA-W) showed higher antioxidant activity due to a higher phenolic and flavonoid content compared to the ethanol extract (TA-E). Quercetin-3-*O*-(2^G^-α-L-rhamnosyl)-rutinoside, quercetin-3-*O*-neohesperidoside, and quercetin are the electron shuttles (ES) identified out of the 11 compounds. TA obtained a 1.39 ± 0.10 amplification factor of power generation that indicates potential bioenergy-generating and associated antiviral characteristic properties. The findings may provide a foundation for developing antiviral medications specifically designed to target virus-related diseases, while minimizing the risk of drug toxicity and reducing the costs of drug development.

## 1. Introduction

For more than 2000 years, traditional Chinese medicine (TCM) has been utilized in the healthcare system for various treatment protocols, diagnostic assessments, and illness prevention. Several applications of TCM include herbal medicine, acupuncture, moxibustion, and other non-medication therapies. Despite its extensive application, debates still rise when TCM and Western medicine are compared based on its treating efficacy. Secondary metabolites found in herbal medicines exhibit various therapeutic effects such as antioxidant, anti-inflammatory, and immunomodulatory properties. Recently, TCM has been reported to contain compounds with electron shuttle (ES)-like structures (e.g., phenolics, anthraquinone, and flavonoid) [1,2]. ESs were specifically classified from the category of antioxidants that were misunderstood in the academic field due to redox-mediating electrons freely resonating within the chemical structure (e.g., the aromatic rings and flavonoids). Their sustainable potential capabilities to mediate resonant electrons within the ESs were not like electrons, that could be consumed for excitation as antioxidants. As recent findings exhibited, these structures are investigated as prospective candidates for the selection of antiviral properties [3]. Hence, this discovery for new effective antiviral medications can aid to bridge the consensus gap between Western medicines and TCM for a wide range of applications.

*Typha angustifolia* L. (TA), commonly referred to as narrow-leaved cattail, is a perennial aquatic herb. It is one of the 30 species classified under the genus *Typha*, which belongs to the *Typhaceae* family [4]. This plant is commonly found in Central Asia, Northern Africa, and North America, specifically in riverbanks, wetlands, and lake shores of tropical regions, growing up to three to seven meters in height [5]. The phytoremediation properties of TA are due to its capacity to store and tolerate high concentrations of various metals [6,7]. Conversely, one of the highly used TCMs to consider is the dried pollen of TA. It has a bright yellow or green appearance, with a diameter that ranges between 19 and 27 µm [8]. TA pollen has been recognized in managing external wounds (e.g., abscesses) and internal bleeding (e.g., dysmenorrhea and dysentery) [9]. Additionally, it promotes urination, relieving conditions like urinary bleeding and strangulation [10]. In traditional medicine, it has been used to treat leprosy [9,11], hyperlipidemia, and to improve microcirculation in China [12]. Beyond its folkloric uses, in vivo studies have demonstrated the anti-inflammatory activity of TA extract in carrageenan-induced rats, showing promising results for treating both acute and chronic conditions [9]. Other studies have further highlighted its pharmacological activities, including immunosuppressive, wound healing, and anti-atherosclerotic effects [13,14,15,16]. Aside from its medicinal properties, TA pollen is used in cooking as a thickener or flour substitute [17]. Despite the numerous investigations on TA pollen, the scarcity of knowledge on compounds containing ES-like structures is still evident. Furthermore, rare research explored the antiviral properties of TA pollen for developing new drugs, utilizing electrochemical analysis through microbial fuel cells (MFCs).

Hsueh et al. [18] demonstrated the bioenergy-generating capabilities of ES compounds with the use of MFC as the platform for electrochemical evaluation. ES (or redox mediators) are functioned to improve the efficiencies of an electron transfer and bioenergy generation to mediate the electron transport chain in cells. Polyphenols derived from natural resources are suitable candidates for ESs. This is due to their low biotoxicity potency, high biocompatibility, and reversible attribute for power-generating capabilities. However, because of the weak acidic nature of polyphenols, it is essential to account for various factors to ensure favorable conditions are observed, including pH levels, the redox potential, and water solubility [18]. Hence, not all plant species that have an abundance of antioxidants which would be directly considered to be ES-plentiful. Furthermore, it was found that herbal extracts containing *o*-diOH and flavonoid compounds play both crucial antioxidant and ES roles which exhibit antiviral activity.

Viral infection symptoms vary from mild to severe, which depends on the immune response and the viral load in the host cells. One of the leading viral diseases in the world is caused by the family Flaviviridae. These are a single-stranded, positive, and encapsulated RNA virus. Dengue virus (DENV), hepatitis C virus (HCV), and Japanese encephalitis virus (JEV) are the only few viruses that belong to Flaviviridae. Both DENV and JEV belong to the genus Flavivirus while HCV belongs to the genus Hepacivirus. Dengue, a vector-borne disease caused by DENV, is transmitted to humans by Aedes mosquitoes. Common symptoms may range from a mild flu-like condition to hypovolemic shock [19]. Japanese encephalitis is transmitted by Culex mosquitoes carrying JEV and is prevalent among individuals residing in rural Asian regions. This disease infects nearby tissues and lymph nodes upon transmission. Common symptoms include abdominal pain and anorexia [20,21]. Conversely, hepatitis C is a bloodborne disease that causes progressive liver damage, culminating in liver cirrhosis and hepatocellular carcinoma. Clinical indications of hepatitis C are fatigue, vomiting, and abdominal pain [22,23,24]. Even though such symptoms mentioned are often typical and can sometimes be overlooked, these diseases are notorious for causing catastrophic infections, leading to high morbidity and mortality rates over the years.

Due to the concerning statistics and life-threatening effects of diverse viral diseases, a critical focus on exploring viable medications is being worked on as a priority to preserve lives worldwide. Recent progress in understanding the electrochemical behavior of herbal medicines has unveiled their potential to possess antiviral properties. Hence, this study aims to identify the bioenergy-generating and ES characteristics of TA pollen. Furthermore, this study aims to identify the bioenergy-generating and electrochemistry-associated characteristics of TA pollen and utilize its electrochemical behavior as a preliminary screening technique to determine its antiviral capability.

## 2. Materials and Methods

### 2.1. Sample Preparation and Extraction

The dried TA pollens were purchased from a local TCM store in Tainan, Taiwan, and authenticated by Dr. Ling-Ling Yang from Taipei Medical University. A voucher specimen (#NIU-TA-001) was deposited at the Department of Chemical and Material Engineering in National I-Lan University. The extraction process followed a 1:20 (g/mL) sample-to-solvent ratio, using 100 g of TA pollen with 2 L of distilled deionized (DD) water or ethanol. The water sample was boiled in a traditional Chinese decoction pot until reduced to 200 mL, while the ethanol sample was extracted using reflux for two hours at 60 °C. Solids were removed by vacuum filtration, and the filtrate was concentrated using a rotary evaporator to obtain crude extracts. The crude extracts were then freeze-dried to achieve a powder-like consistency.

### 2.2. Phytochemical Analysis

To further evaluate electrochemical characteristics, total polyphenols (TP), flavonoid (TF), and condensed tannin (TCT) content were quantitatively determined with the use of water extracts (TA-W) and ethanol extracts (TA-E).

#### 2.2.1. Total Phenolic Content (TPC) Analysis

A gallic acid-based standard solution (1 mg/mL) and sample solution (10 mg/mL) were used to determine TPC of TA pollen with minor modifications from Tsai et al. [3]. The solution was serially diluted by two-fold to obtain seven concentrations (1000 to 15.6 μg/mL). Then, 20 µL of each solution was combined with 100 µL of 0.2 N Folin–Ciocâlteu reagent and 80 µL of 7.5% Na_2_CO_3_. After a 30 min reaction, 200 µL aliquots were placed in a 96-well plate in triplicates for accuracy. The absorbance was measured at 600 nm using an ELISA microplate reader (Multiskan SkyHigh, Thermo Scientific, Waltham, MA, USA).

#### 2.2.2. Total Flavonoid Content (TFC) Analysis

A rutin-based standard solution (1 mg/mL) and sample solution (10 mg/mL) were used to determine the TFC in TA pollen, following a modified version of Tsai et al. [3]. Each 500 μL standard solution was reacted with 500 μL of 2% AlCl_3_ for 15 min. Then, 200 µL aliquots were transferred in 96-well plate. Then, the absorbance of the mixture was measured at 430 nm.

#### 2.2.3. Total Condensed Tannin (TCTC) Analysis

A catechin-based standard solution (1 mg/mL) and sample solution (10 mg/mL) were used to determine the TCTC of TA pollen, following the method of Tsai et al. [3]. For this, 300 µL of solution was reacted with 600 µL of vanillin in 80% H_2_SO_4_ and incubated in a water bath at 50 °C for 15 min. Then, 200 µL aliquots were transferred in a 96-well plate and the absorbance was measured at 530 nm.

### 2.3. Antioxidant Activity

Antioxidant activity of TA samples was determined using the 2,2-diphenyl-1-picrylhydrazyl (DPPH) free radical scavenging activity and ferric-reducing antioxidant power (FRAP) assays.

#### 2.3.1. DPPH Free Radical Scavenging Activity

A 200 µM DPPH solution was prepared as the reaction reagent. A standard solution (1 mg/mL) was made by dissolving 10 mg of ascorbic acid in 10 mL of ethanol [3]. It was followed by two-fold serial dilutions to achieve concentrations from 1000 µg/mL to 7.8 µg/mL. Then, 50 µL of each sample (10 mg/mL) and standard solution was reacted with 150 µL of DPPH solution. The absorbance was measured at 517 nm. The radical scavenging activity (%RSA) was calculated using Equation (1), and linear regression was applied to estimate the half maximal inhibitory concentration (IC_50_) at 50% RSA.

(1) $$\%RSA=\left[1-\frac{\left(A_{sample}-A_{blank}\right)}{\left(A_{control}-A_{blank}\right)}\right]\times 100$$

%RSA = % Radical Scavenging Activity

A_sample_ = absorbance of sample

A_control_ = absorbance of control

A_blank_ = absorbance of blank

#### 2.3.2. Ferric Reduction Antioxidant Power (FRAP) Assay

A FRAP reagent was prepared using a 10:1:1 ratio of pH 3.9 acetate buffer, 5 mM 2,4,6-Tripyridyl-S-triazine (TPTZ) solution, and 20 mM FeCl_3_∙6H_2_O solution, following a method from Tsai et al. [3]. For this, 2 mg/mL Trolox stock solution was made by dissolving 10 mg of Trolox in 5 mL of a 2:3 ethanol-DD water mixture. Two-fold serial dilutions were performed to obtain concentrations from 2000 µg/mL to 31.3 µg/mL. For the reaction mixture, 50 µL of each solution was mixed with 1450 µL of FRAP reagent. Then, 200 µL aliquots were transferred to a 96-well plate. The absorbance was measured at 593 nm.

### 2.4. High-Performance Liquid Chromatography (HPLC) Analysis

In conducting the high-performance liquid chromatography (HPLC) analysis, Hypersil™ BDS C18 column (250 × 4.6 mm, 5 µm, Thermo Fischer Scientific Inc.), a 10 mg/mL ethanol extract, was prepared. It was filtered using a 0.45 μm syringe filter, with 1 mL transferred into each vial. A Shimadzu HPLC model LC-2050C 3D was used to separate and identify the major compounds in TA pollen. The chromatographic analysis utilized Mobile Phases A (water), B (acetonitrile), and C/D (methanol). The column oven was maintained at 40 °C with a flow rate of 1 mL/min. The gradient program started at 0 min with 95% Mobile Phase A and 5% Mobile Phase B, reaching 100% Mobile Phase B at 60 min [3].

**Figure 1:**
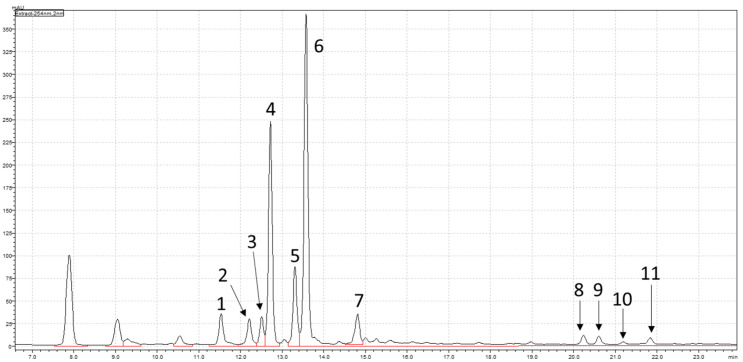
Chromatographic fingerprint of EtOH extract of TA pollen from 2D HPLC-PDA at 245 nm.

HPLC analysis identified the bioactive compounds present in TA-E. The peaks from the chromatographic fingerprint (Figure 1) were analyzed and compared to reference compounds from Tao et al. [25] for validation. The analysis identified two major compounds, isorhamnetin-3-*O*-neohesperidoside (I3ON) and typhaneoside, based on their peak intensity, area, and retention time. These compounds were also confirmed as the primary flavonoids in TA pollen, as reported in the previous literature [26].

Figure 1 shows the peaks displayed on the chromatographic fingerprint of TA-E from 2D HPLC-PDA. The following compounds detected include: (1) quercetin-3-*O*-(2^G^-α-L-rhamnosyl)-rutinoside; (2) quercetin-3-*O*-neohesperidoside; (3) kaempferol-3-*O*-(2^G^-α-L-rhamnosyl)-rutinoside; (4) isorhamnetin-3-*O*-(2^G^-α-L-rhamnosyl)-rutinoside (typhaneoside); (5) kaempferol-3-*O*-neohesperidoside; (6) isorhamnetin-3-*O*-neohesperidoside; (7) isorhamnetin-3-*O*-rutinoside; (8) quercetin; (9) naringenin; (10) kaempferol; and (11) isorhamnetin.

### 2.5. Power-Density Determination via MFCs

#### Microbial Fuel Cell Framework and Microbial Cultures

An H-type double chamber (DC) MFC was used to assess the bioenergy generation of TA pollen. Graphite anode and cathode electrodes (Grade: IGS743; Central Carbon Co., Ltd., Taipei, Taiwan) were soaked in electrolyte and culture broth, with an area of 1.649 cm^2^. A 200 mL operating volume was isolated by a proton exchange membrane (DuPont™ Nafion^®^ NR-212, Wilmington, DE, USA) with an immersed area of 0.000452 m^2^ in each chamber. In the cathodic chamber, the 6.38 g K_3_Fe(CN)_6_ and 17.42 g K_2_HPO_4_ electrolyte was dissolved in 200 mL deionized water. The anodic chamber contained a culture broth prepared with *Aeromonas hydrophila* (National Center for Biotechnology Information or NCBI ID = 644), a Gram-negative, electroactive bacterium that was selected for its ability to degrade organic compounds and form biofilm on electrodes, enhancing electron transfer [27]. The bacteria were precultured in 100 mL LB broth (Difco™ LB Broth, Miller; Luria-Bertani, Minato, Japan) at 30 °C and 125 rpm for 12 h. Then, 1 mL of the precultured broth was added to 18 flasks, each with 100 mL sterilized LB broth, cultured until optical density reached 2.1 at 600 nm. The anodic chamber was supplied with 200 mL of culture broth, and a 1.2 g of TA sample was dissolved in 12 mL of deionized water and ethanol. Power density was measured with TA concentrations of 500 mg/L, 1000 mg/L, and 2000 mg/L. The chamber and electrode were rinsed with sterile deionized water after each concentration. The optical density was maintained at 2.1, with Blank 1 used before the 500 mg/L sample and Blank 2 after the 2000 mg/L sample for consistency. Lastly, 0.1 mL of 0.6 M dopamine was used as a standard after Blank 2.

### 2.6. Power Density 

The power generation of MFCs supplemented with TA extracts was tested using a D/A system (DAS 5020; Jiehan Tech Corp., Taichung City, Taiwan) to measure voltage and electric current over time. For consistency, the external resistance of the MFC was set to 1 kΩ [3]. Power and current densities were calculated using standard equations:

(2) $$P_{density}=\frac{V_{MFC}\times I_{MFC}}{A_{anode}}$$

(3) $$I_{density}=\frac{I_{MFC}}{A_{anode}}$$

The working area of the graphite anode, A_anode_, was used for calculations. The voltage of the highest power point (V_MFC_) and current of the highest power point (I_MFC_) were identified through Linear Sweep Voltammetry (LSV) (Jiehan 5600, Jiehan Technology Corp., Taichung City, Taiwan). The LSV plot was used to determine V_MFC_ by locating the highest current–voltage product, followed by identifying I_MFC_ at the same point.

### 2.7. Statistical Analysis

In the phytochemical analysis (Section 3.1) and antioxidant activity (Section 3.2), the tabulated results were displayed as average ± standard deviation from the three replicates. Analysis of Variance (ANOVA) was used to analyze the readings, employing a significance level of *p <* 0.05 using Microsoft Excel© v16.0 software. Descriptive statistics were used to assess the variability of the parameters.

## 3. Results and Discussion

### 3.1. Total Phytochemical Analysis

Table 1 summarizes all the data obtained from the phytochemical analysis. With a determination coefficient (R^2^) of more than 0.90, the calibration curves explicitly demonstrated a promising linear relationship between the signal and the concentration of the reference standards. This implies that all the polyphenols, flavonoids, and condensed tannins found in the samples were within QA/QC ranges. TP generated a standard curve with an equation of y = 5.367x − 0.0014 (R^2^ = 0.9992) expressed in gallic acid equivalents (GE). TF obtained a linear equation of y = 27.533x + 0.0459 (R^2^ = 0.9999) expressed in rutin equivalents (RE). TCT generated the linear equation y = 7.2500x + 0.3034 (R^2^ = 0.9944) expressed in catechin equivalents (CaE). TA-W had a higher TP and TF content than TA-E, obtaining 32.6019 ± 0.0404 mg/g and 31.4541 ± 0.0208 mg/g, respectively. Conversely, the TCT content of TA-E was higher with a value of 44.2746 ± 0.1122 mg/g (ca. 2.79 folds to TA-W). This suggests that water is a somewhat more favorable solvent in extracting the phenolic and flavonoid content. In contrast, an ethanol solvent is preferably utilized in achieving an optimal amount of condensed tannin extract.

Phytochemicals play a significant role in the health advantages of plant-based diets due to their pharmacological properties. They are a class of diverse compounds that fall into several groups such as phytosterols, polyphenols, flavonoids, carotenoids, and organosulfur compounds [28]. Phenolic compounds are recognized to possess antioxidant properties. Its redox potential assists to function as chelating agents of metal ions or hydrogen donors. High stability is conferred based on the radical produced during the radical neutralization process by an *ortho* configuration of hydroxyl groups. Additionally, greater antioxidant contents are reflected in a higher amount of hydroxyl groups [29,30]. Hsueh et al. [18] discussed that polyphenols are found to act as an ES where it can be reversibly expressed for the bioenergy-generating capacity under favorable conditions. In addition, it is essential to consider various factors (e.g., concentration, pH level, solubility, and biotoxicity) as they can affect the reduction potential of polyphenol compounds.

The chemical structure of polyphenols can be categorized based on the presence of several phenolic groups, classified as flavonoids and non-flavonoids. Plants are omnipresent sources of flavonoids, a broad class of polyphenolic chemicals with a benzoyl-γ-pyrone structure [31]. Aside from this, a flavonoid’s bioactivity is determined by its structural substitution patterns in its C6-C3-C6 rings, which serves as the basic skeleton [32]. Flavonoids are abundant in human diets and are recognized in combating various viral infections through multiple stages such as viral entry, replication, and protein translation [33]. On the other hand, condensed tannins are polymers that are developed from two to more leucoanthocyan flavan-3,4-diols [34].

Numerous plant-based polyphenols have been identified and are becoming more significant in the modification of antiviral drugs due to their inexpensive production, accessibility, and minimal side-effects. In addition, negative impacts from viruses are not limited to its replication, rather how the immune system responds to infections, including oxidative stress and inflammation. Thus, combining both antioxidant and antiviral medication could exhibit more potent effects for treating viral infections and counteracting the emergence of antiviral resistance [35,36]. Based on the results obtained, the phenolic, flavonoid, and condensed tannin contents reflect on the prospective health benefits that are found in TA. This only applies to how TA can be a prospective candidate for the in-depth assessment and clinical research of drug development on various viral diseases such as dengue fever, hepatitis C, and Japanese encephalitis. Also, the results show that using different solvents can vary the optimal amount of phytochemical content extraction.

### 3.2. Antioxidant Activity Assessment

The antioxidant activity was reported to be correlated to many disease-resisting capabilities. Thus, revealing such activities was of great importance in herbal medication. As shown in Table 2, the standard curve generated in doing the DPPH scavenging activity is y = 2731.35x − 4.3973 with a determination coefficient (R^2^) of 0.9981, expressed as an ascorbic acid equivalent. Ascorbic acid is used as a positive control in the said assessment, with an IC_50_ value of 0.0199 ± 0.0004 mg/mL. The FRAP assay generated a standard curve with a linear equation of y = 2.2024x + 0.1548 with R^2^ = 0.9919, as expressed in the Trolox equivalent. Comparing the values obtained from the crude extracts, TA-W has a lower IC_50_ value (0.8784 ± 0.0117) and higher FRAP assay value (35.2568 ± 0.0145). On the other hand, TA-E obtained an IC_50_ value and FRAP assay value of 2.4073 ± 0.1091 and 25.9639 ± 0.0167, respectively. Both TA-W and TA-E were higher compared to ascorbic acid. However, TA-E was obtained to have a greater difference with ascorbic acid.

Antioxidants found in plants have received considerable attention because of their ability to prevent damage caused by oxidative stress. Oxidative stress happens when there is an increase in the reactive oxygen species (ROS) level from ROS formation and detoxification. This process will then lead to a disturbed cellular function that causes the development of life-threatening diseases [37,38]. Generally, antioxidants follow two mechanisms in scavenging free radicals: by donating hydrogen to generate stable compounds, and by reducing any compounds (i.e., metals, carbonyls, free radicals) by transferring an electron [38]. In this study, the DPPH radical scavenging assay and FRAP assay were conducted as in vitro analyses to quantitatively determine the antioxidant properties of TA pollen. The IC_50_ value obtained in the experiment dictates the concentration of antioxidants needed to decrease the initial DPPH concentration by 50%. The solution has a purple hue and develops into a colorless or yellow color when neutralized by radical scavengers. Hence, the lower IC_50_ interprets greater antioxidant activity. In contrast, the FRAP assay measures the capacity of antioxidants in reducing ferric ions under acidic conditions. Antioxidants are assessed based on their capability to reduce Fe(III)/tripyridyltriazine complexes. This requires a higher FRAP assay value which indicates a higher level of antioxidant activity. Between the DPPH free radical scavenging activity of TA-W and TA-E, it reveals that water is a more appropriate solvent in achieving the maximum antioxidant activity from TA pollen. Based on its phytochemical analysis, phenolic and flavonoid compounds made a significant contribution to the antioxidant activity of TA-W. This is because TA-W has a lower IC_50_ value and a higher phenolic and flavonoid content.

### 3.3. HPLC Analysis of T. angustifolia Extract

To exhibit the efficacy of medicinal herbs, showing the presence of such effective compositions is of great significance via HPLC analysis. 

The diverse pharmacological effects (e.g., the synergistic interaction of disease treatment) attributed to TCM stem from their bioactive components like phenols, flavonoids, tannins, and phenolic acids. This section provides several therapeutic effects of the bioactive compounds detected in the TA extract that are also present in other herbal and dietary plants. For example, quercetin and kaempferol are under the subgroup flavonols. Flavonols are commonly found in significant quantities in plant sources, typically in glycoside forms. They derive from 3-hydroxyflavone backbone, and are often linked with a ketone group [39,40]. Apart from their antioxidant properties, prior research indicated that both compounds have the potential to relieve symptoms associated with Alzheimer’s disease (AD). Furthermore, they suggested that a combination of herbal extracts (e.g., *Camellia sinensis* L., *Cyperus rotundus* L., and *Gingko biloba* L.) abundant in quercetin and kaempferol could address AD-related brain disorders [41]. Both compounds have also emerged with a potential to possess antihypertensive, anti-atherosclerotic, and cardioprotective effects which makes this a potential treatment for cardiovascular disease [42,43]. Stemming from the same subgroup, isorhamnetin originates as a quercetin molecule that has undergone methylation. Isorhamnetin extracted from *Hippophae rhamnoides* L. showed potent anti-tumor activity. Moreover, other research suggested that it promotes the carbohydrate metabolism and exhibits anti-diabetic activities [44,45]. Another constituent found within the TA extract is kaempferol-3-*O*-neohesperidoside, categorized as a flavonoid glycoside and belonging to the family of flavonoid derivatives. The said compound is also present in *Dypsis pembana* which exhibits the highest antidiabetic activity that inhibits alpha glucosidase enzymes in vitro and in silico [46]. On the other hand, *Phoenix dactylifera* L. extracts contain substances such as phenolics and flavonoids (flavanones, isoflavanones, andfava-3-ols) with antibacterial and antiviral properties, which was found with the use of GC-MS analysis [47]. In fact, polyphenolics have also been used in the food industry as they possess antimicrobial activity. *Punica granatum* peel extracts were found to contain flavonoids that acted as reducing and capping agents [48]. Other derivatives of isorhamnetin that were also detected are isorhamnetin-3-*O*-rutinoside and isorhamnetin-3-*O*-neohesperidoside. These compounds own the antioxidant and wound healing properties of the dried flowers of *Calendula officialis* L. [49,50]. Furthermore, naringenin, categorized within the flavanone subgroup, is present in TA pollen. Flavanones, aromatic and colorless ketones derived from flavones, are commonly distributed in various *Citrus* species (e.g., lemon, orange, and clementine). Furthermore, they are known to possess diverse biological activities such as antitumor, antiviral, antibacterial, and anti-inflammatory effects [51,52]. Meanwhile, typhaneoside from *Typha orientalis* L. was also reported to alleviate the symptoms of non-alcoholic fatty liver disease (NAFLD) due to its resistance to glucose and lipid metabolism disorders [53].

As shown, all identified bioactive compounds were polyphenols, specifically defined as flavonoids. It was mentioned earlier that polyphenols obtained from natural resources were bioenergy-generating and electron-medicating. This is due to the chemical characteristics (i.e., aromaticity, nature of substituent, and its positions) found in organic ESs that are also inherent in polyphenols [18]. The chemical configuration of an ES significantly impacts the stability and dispersion of electrons and radicals, which is crucial for bioenergy extraction.

### 3.4. Power Density Measurements

**Figure 2:**
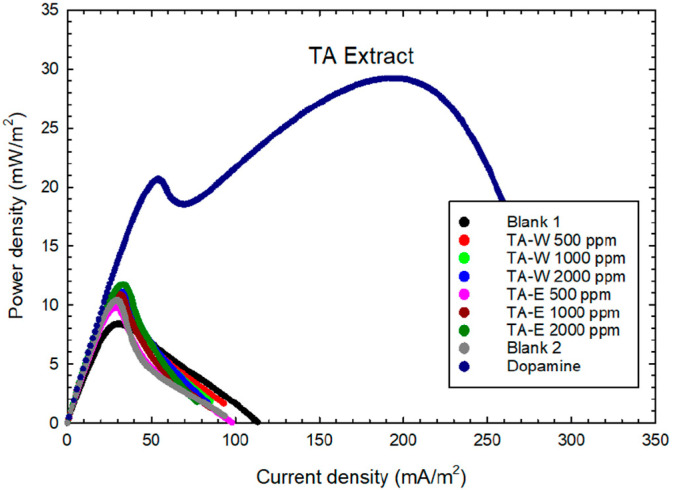
Power density profile of TA pollen water and ethanol extracts.

In fact, such electron-transferring potential of test herbal extracts should be directly reflected upon bioelectronic-generating characteristics via MFC platforms. Table 3 presents the power-generating performance and its corresponding amplification factor. It shows that the samples were assessed using varying concentrations (i.e., 500 mg/L, 1000 mg/L, and 2000 mg/L). The result reveals that TA-E with 2000 mg/L obtained the highest power density (11.71 ± 0.34 mW/m^2^) and amplification factor (1.39 ± 0.10) among all the samples. As shown in Figure 2, the top rank power density of pure chemical dopamine was adopted as a reference standard and control in the system. It is anticipated for chemical mixture-bearing herbal extracts to obtain lower values of the power density.

MFCs are bio-electrochemical systems that utilize microorganisms in converting organic matter into electricity. Furthermore, the microorganism (bacteria) oxidizes the organic matter and moves into an electrode that generates an electric current. MFCs are commonly utilized in treating wastewater as they offer a sustainable and energy-efficient method to provide clean water. Recently, new applications of MFCs were observed that can detect phytochemicals with ES-like structures may possess antiviral properties. Chen et al. [54] identified that the disease-treating capabilities of herbal medicines is not only exclusively determined by their antioxidant activity; instead, it also involves their electrochemical catalytic features. ES compounds display electrochemically stable intermediates during redox reactions, ensuring their resilience over successive electrochemical activities. In contrast, non-renewable antioxidant compounds scavenge free radicals but lack the ability to form stable intermediates. As a result of the irreversible oxidation, these compounds break down over long periods of electrochemical activity, making them not appropriate as sustainable catalysts [18]. In contrast, ES compounds serve as catalysts that remain stable and intact during metabolic processes, enhancing both the effectiveness and stability of drug treatments. The distinction between antioxidant and ES compounds plays a critical role in determining the therapeutic efficacy. Given the pivotal function of ES compounds in medication, as opposed to antioxidants, they hold greater potential as promising therapeutic agents.

When a plant extract demonstrates a power density ranking with an amplification factor exceeding ca. 2.0 concerning a blank, it signifies that the extract has attained a satisfactory level of electron transfer potential [3]. This observation suggests a competitive antiviral characteristic within the plant extract. The results suggest that the power density values report the electrochemical potential of TA. It is observed that it exhibited a slightly low value. Relating the results from Section 3.3, it can be hypothesized that only few compounds exhibited electrochemical activity while others did not, indicating a low bioenergy content. This result could also have an impact on its antiviral capabilities. A previous study [55] investigated and compared the raw from being put through several processing methods (i.e., stir-frying, soaking in wine or vinegar, and high-pressure steaming) of extracting the phytochemicals in *Rheum palmatum*. It revealed that different methods lead to variations in its chemical profile, influencing its bioactivity. Furthermore, stir-frying with vinegar was found to be the most appropriate method as it yielded the highest total phenolic contents, which also exhibited an increase in the amplification factors of the power density.

The increase in the antioxidant activity suggests that herbal extracts have the potential to acquire bioenergy-generating characteristics. Such findings indicated that the extracts could also persist as electrochemical catalysts in prolonged bioactivities. However, a presence of high antioxidant levels and low electron-stimulating capabilities are observed, where biological activities (e.g., antiviral) tend to occur briefly. This occurrence results in being not highly effective in preventing viral infections [3,54]. In addition, the electrochemical characteristics to be revealed as either antioxidant or ESs were also strongly influenced by the chemical structure and environmental settings. *Ortho*- and *para*-polyhydroxy substituents attached in aromatic rings could exhibit reversible and stable ES capabilities. Through inductive and resonance effects, the position of the substituents (such as *para*, *ortho*, and *meta*) on an aromatic ring influences the reactivity and regioselectivity of electrophilic (or nucleophilic) aromatic substitutions. On the other hand, flavonoid compounds, specifically those with polyhydroxy substituents in *ortho* positions, could enhance antiviral properties through synergistic effects. Considering the pathology, viral-induced oxidative stress can disrupt the host’s redox balance, leading to the weakening of immune system. Consequently, bioactive compounds that are abundant in antioxidants emerge as prospective candidates for combating viral infections. Hence, the effectiveness of flavonoids for antiviral activity are linked to their antioxidant and electron-mediating properties [18,56].

## 4. Significance of the Study

This study highlights the significance of different prospectives in shaping the pharmacological and biological properties of TA pollen. By combining the results from extraction techniques, a connection is established for assessing both the phytochemical composition and bioenergy-producing capabilities of TA pollen. Additionally, the findings indicate its potential as a promising candidate for treating serious viruses, such as dengue fever, hepatitis C, and Japanese encephalitis. Symptoms of these diseases could emerge from asymptomatic to fever, body aches, and joint pains. Neurological manifestations (e.g., seizures and convulsions) were also observed in affected children [57]. Diseases resulting from Flaviviridae exhibit severe effects since it primarily targets the central nervous system. This makes the treatment more challenging due to its impact on the immune system. People often resort to self-medication to address symptoms due to poverty, limited access, and a lack of awareness [58]. However, improper self-medication may exacerbate the condition which may result in fatal complications. Therefore, herbal medicines like TA pollen are valuable for their low cost and accessibility. Initial studies on this herbal medicine demonstrated a range of physiological effects, especially concerning blood circulation.

The results revealed that TA-W contains a higher phenolics and flavonoids content, making it the preferred solvent for the optimal extraction of these phytochemicals. In contrast, TA-E demonstrated promising ES capabilities, as evidenced by both its highest power density value (at 2000 mg/L) and the observed augmentation in its power density with an increasing concentration. Nevertheless, these values were comparatively less substantial when compared with those obtained from TA-W. In fact, the amplification factors did not surpass the 2-fold basis with respect to the blank sample, reducing the effectiveness compared to the previous studies with different plant samples [27,54]. This finding can be caused by compounds detected from the HPLC analysis. It was mentioned earlier that naturally obtained polyphenols are manifested to possess either or both antioxidant and bioenergy-generating characteristics. The electrochemical activities are likely dominated by antioxidant effects due to the selection of conditions that may be slightly less than ideal, resulting in a relatively lower bioelectricity-stimulating performance. Here, quercetin and its derivatives (quercetin-3-O-(2G-α-L-rhamnosyl)-rutinoside and quercetin-3-O-neohesperidoside) were the only ES compounds found in the sample. The primary aim of our research is to evaluate the antiviral activity of *Typha angustifolia* pollen (TA) during the initial screening follow-up studies to explore the interaction mechanisms between TA compounds and viral target proteins, with the goal of developing new antiviral drugs being inevitably required.

## 5. Conclusions

The research highlights the potential of TA pollen as a promising candidate in the field of drug discovery and development for illnesses induced by viral pathogens. It was found that TA-E at 2000 mg/L showed the highest power density via MFC, indicating potential for bioenergy stimulation and electron transport. This innovative electrochemical method is employed to screen potential herbal remedies with an antivirus property. The findings potentially lay the groundwork for the development of antiviral medications tailored to target diseases associated with viruses, while reducing the risk of drug toxicity. Follow-up exploration is still recommended to assess additional crucial parameters contributing to the continued advancement of effective drug development.

## Figures and Tables

**Table 1:** Total phytochemical content of *T. angustifolia* extracts.

Sample	Total Phenolic Content (mg GAE per g CE)	Total Flavonoid Content (mg RE per g CE)	Total Condensed Tannin Content (mg CE per g CaE)
TA-W	32.6019 ± 0.0404	31.4541 ± 0.0208	15.8784 ± 0.0361
TA-E	24.4638 ± 0.0289	27.3874 ± 0.0361	44.2746 ± 0.1122
Standard curve	y = 5.367x − 0.0014 (R^2^ = 0.9992)	y = 27.533x + 0.0459 (R^2^ = 0.9999)	y = 7.2500x + 0.3034 (R^2^ = 0.9944)

GAE, gallic acid equivalent; RE, rutin equivalent; CaE, catechin equivalent; CE, crude extract; TA-W, *T. angustifolia* water extract; TA-E, *T. angustifolia* ethanol extract.

**Table 2:** Antioxidant assessment of *T. angustifolia* water and ethanol extracts.

Samples	DPPH IC_50_ (mg per mL)	FRAP (mg TE per g CE)
TA-W	0.8784 ± 0.0117	35.2568 ± 0.0145
TA-E	2.4073 ± 0.1091	25.9639 ± 0.0167
Ascorbic acid	0.0199 ± 0.0004	-
Standard curve	y = 2731.35x − 4.3973 (R^2^ = 0.9981)	y = 2.2024x + 0.1548 (R^2^ = 0.9919)

TE, Trolox equivalent; CE, crude extract; TA-W, *T. angustifolia* water extract; TA-E, *T. angustifolia* ethanol extract.

**Table 3:** Power density analysis and amplification factor of *T. angustifolia* water and ethanol extracts at different concentrations. Amplification factor with respect to 1.

Samples	Power Density (mW/m^2^)	Amplification Factor
Blank 1	8.39 ± 0.36	1
TA-W 500 mg/L	10.72 ± 0.59	1.27 ± 0.12
TA-W 1000 mg/L	10.78 ± 0.56	1.28 ± 0.12
TA-W 2000 mg/L	11.11 ± 0.52	1.32 ± 0.12
TA-E 500 mg/L	9.80 ± 0.40	1.17 ± 0.10
TA-E 1000 mg/L	10.90 ± 0.83	1.30 ± 0.15
TA-E 2000 mg/L	11.71 ± 0.34	1.39 ± 0.10
Blank 2	10.41 ± 0.40	1.24 ± 0.10

TA-W, *T. angustifolia* water extract; TA-E, *T. angustifolia* ethanol extract.

## Data Availability

Data are contained within the article.
